# The Enolase of the *Haemophilus influenzae* Mediates Binding to Collagens: An Extracellular Matrix Component

**DOI:** 10.3390/ijms242115499

**Published:** 2023-10-24

**Authors:** Yesenia Osorio-Aguilar, Maria Cristina Gonzalez-Vazquez, Patricia Lozano-Zarain, Ygnacio Martinez-Laguna, Lidia Baylon-Pacheco, Jose Luis Rosales-Encina, Alejandro Carabarin-Lima, Rosa del Carmen Rocha-Gracia

**Affiliations:** 1 Posgrado en Microbiología, Laboratorio de Microbiología Hospitalaria y de la Comunidad, Centro de Investigaciones en Ciencias Microbiológicas, Instituto de Ciencias, Benemérita Universidad Autónoma de Puebla, Puebla 72570, Mexico; yesenia.osorioa@correo.buap.mx (Y.O.-A.); patricia.lozano@correo.buap.mx (P.L.-Z.); ygnacio.marinez@correo.buap.mx (Y.M.-L.); 2 Licenciatura en Biotecnología, Instituto de Ciencias, Benemérita Universidad Autónoma de Puebla, Puebla 72570, Mexico; maria.gonzalezvazquez@viep.com.mx (M.C.G.-V.); alejandro.carabarin@correo.buap.mx (A.C.-L.); 3 Departamento de Infectómica y Patogenesis Molecular, CINVESTAV-IPN, Avenida Instituto Politécnico Nacional No. 2508, Colonia San Pedro Zacatenco, Delegación Gustavo A. Madero, Mexico City 07360, Mexico; lbaylon@cinvestav.mx (L.B.-P.); rosales@cinvestav.mx (J.L.R.-E.)

**Keywords:** *Haemophilus influenzae*, enolase, collagen-binding protein

## Abstract

Enolase proteins play a significant role as moonlighting proteins. In their role as surface-associated enolase, they have multiple functions as they interact with extracellular matrix proteins. Type I and III collagens are the major constituents of this extracellular matrix, and collagen is one of the targets of interaction with the enolase of many pathogens, thereby helping the colonization process and promoting the subsequent invasion of the host. This work aimed to determine the participation of non-typeable *H. influenzae* enolase as a collagen-binding protein. In this study, through the use of in vitro tests it was demonstrated that recombinant enolase of non-typeable *H. influenzae* (rNTHiENO) strongly binds to type I collagen. Using molecular docking, the residues that could take part in the interaction of non-typeable *H. influenzae* enolase-type I collagen (NTHiENO-Cln I) and non-typeable *H. influenzae* enolase-type III collagen (NTHiENO-Cln III) were identified. However, in vitro assays show that NTHiENO has a better affinity to interact with Cln I, concerning type Cln III. The interaction of NTHiENO with collagen could play a significant role in the colonization process; this would allow *H. influenzae* to increase its virulence factors and strengthen its pathogenesis.

## 1. Introduction

*Haemophilus influenzae*, a pleomorphic Gram-negative coccobacillus, is a common commensal of the upper respiratory tract [1]. It is a human pathogen capable of causing severe invasive diseases, including meningitis, septicemia, and pneumonia. *H. influenzae* strains are grouped based on the presence or absence of capsular polysaccharides; there are six encapsulated serotypes (a, b, c, d, e, and f), and non-encapsulated strains are called non-typeable *H. influenzae* (NTHi) [1]. Since the introduction of the vaccine against *H. influenzae* serotype b in the 1990s, the burden of related infections has been principally dominated by NTHi [2].

Infections produced by NTHi are a major cause of exacerbations of chronic obstructive pulmonary disease (COPD) in adults. Exacerbations of COPD and otitis media are the most prevalent infections caused by NTHi. These infections result in missed time from work, emergency room visits, hospitalizations, and respiratory failure [3].

NTHi survival in the host is multifaceted due to several virulence factors involved in complement resistance, biofilm formation, modified immunoglobulin responses, and utilization of the host’s proteins as a secondary strategy for increasing the adherence ability [2]. The host–pathogen interactions through adhesins of NTHi have not been fully investigated. Nonetheless, the persistence mechanisms of NTHi include the expression of multiple redundant adhesins that mediate binding to host and (ECM) [3]; therefore, the relationship between adhesins and the ECM had an important role in the success of NTHi colonization and virulence [4].

Currently, moonlight proteins are multifunctional proteins, i.e., capable of performing multiple physiologically relevant biochemical or biophysical functions [5]; some functions of moonlighting proteins include adhesion to host epithelial cells, mucus, and extracellular matrix components [6,7]. 

It is generally postulated that moonlighting proteins indirectly support pathogen binding to host cells, thereby facilitating the colonization process and promoting the subsequent invasion of the host organism [8]. 

The enolase (phosphopyruvate hydratase E.C. 4.2.1.11) plays a prominent role as a moonlighting protein [9]; this is a metalloenzyme that catalyzes the reversible conversion of 2-phospho-D-glycerate (2-PGE) to phosphoenolpyruvate in the glycolysis pathway, and therefore, it is one of the most abundantly expressed proteins in the cell cytoplasm of many organisms [10,11]. This enzyme belongs to a class of surface proteins that do not possess classical machinery for its transport to the cell surface [11].

The surface-associated form of enolase has multiple functions in bacterium–host interactions [12]. The most common enolase moonlighting function appears to be binding to plasminogen (Plg) [9], but it has also been found as a cell surface adhesin in a variety of microorganisms interacting with the ECM proteins, such as laminin [13], fibronectin [14], collagen [12], among other; these proteins are important components of basement membranes and extracellular matrices in tissue barriers [9]. 

The most abundant family present in ECM is collagens [15]. To date, 28 different types (from I to XXVIII) have been identified [16]; of these, collagens I to V are the most encountered [17], and type I collagen is the most abundant in the human body [18]. 

The triple helix of type-I collagen is composed of two α1(I) chains and one α2(I) chain [19], while type III collagen is a homotrimer of three identical polypeptide chains [20] supercoiled around each other in a right-handed triple helix [21].

The typical amino acid sequence of collagen is Gly-XY, in which X and Y can be any amino acid; however, the X-position is occupied almost exclusively by Pro, whereas Hyp is found predominantly in the Y-position [22,23], Glycine, proline, and hydroxyproline (Hyp) contribute to 57% of total amino acids (AAs) in collagen [24]. Collagen is known to have tens of putative interaction partners, including extracellular matrix glycoproteins, such as fibronectin and decorin, and also cell surface receptors, such as integrins and the consensus motif can be formulated as triple-helical GXX’GER, where X’ is often 4-Hyp, The GFOGER (O = 4-Hyp) is a widely studied site, while other similar motifs include GROGER, GLOGER, GMOGER, GLSGER, GQRGER, among others [23].

In multiple studies, it has been reported that among the components of the ECM, collagen is one of the targets of interaction with the enolase of many pathogens and some prebiotic bacteria [9,25,26], which were identified in the region from 73 to 140 amino acid residues of *Lactobacillus plantarum* enolase as being important domain to contact and binding to type I collagen [27].

On the other hand, for the case of *H. influenzae* enolase, based on our knowledge, there are no reported data about the interaction between enolase–collagen. Therefore, in this study, we investigated the ability of non-typeable *H. influenzae* enolase (NTHiENO) to bind to type I and III collagen via molecular docking and in vitro assays. All results show that enolase of NTHi is capable of binding with type I collagen.

## 2. Results

### 2.1. Modeling of NTHiENO Dimeric Structure

**Figure 1:**
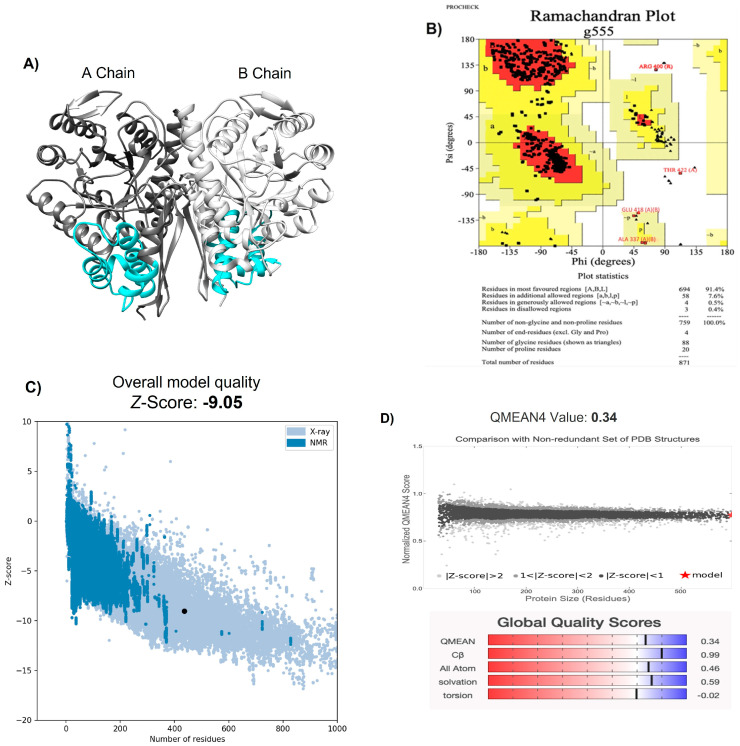
Structural modeling of the *Haemophilus influenzae* enolase dimer. (**A**) NTHiENO dimeric structure obtained via SWISS-MODEL and visualized using Chimera: secondary structure depictions are shown in alpha helices and beta strands. The dark grey and light grey colours represent the A chain and B chain, respectively (both have the same sequence), while the cyan colour represents the region of putative binding to collagen (72–139), which is equivalent to amino acid residues 73–140 of *Lactobacillus plantarum* enolase, identified as region collagen type I binding. (**B**) Ramachandran plot analysis of the theoretical model of NTHiENO dimeric. All residues except Gly and Pro are shown as square dots located in the most favored regions (91.4% in the red area), additional allowed regions (7.6% in the dark yellow area), generously allowed regions (0.5% in the light-yellow area) and disallowed regions (0.4% in the white area). (**C**) ProSA analysis (the black dot represents the model z-score (−9.05) for the protein of NTHiENO. (**D**) Normalized QMEAN score graphic showing the z-score value and the position of the model of NTHiENO (red star) in the set of PDB structures used for evaluation. The Global Quality Scores are shown below: red (worse) and blue (better).

Initially, the homology modeling of the NTHiENO dimer (GenBank: MF405339) was constructed using the SWISS-MODEL platform [28], and the structure obtained was visualized with the Chimera program [29] (Figure 1A). As a template, the crystallized homo dimer of *E. coli* enolase (PDB ID 6BFY) was used with a resolution value of 1.81 Å [30] and showed 85.61% of identity with NTHiENO. The modeled structure was analyzed using PROCHECK; the Ramachandran plot shows values of 91.4% for the most favored regions, 7.6% for additional allowed regions, 0.5% for generously allowed regions, and 0.4% for disallowed regions (Figure 1B), thereby the values indicating that the mayor conformations of the amino acid residues are within the most favored or allowed regions and that the stereochemical parameter Phi–Psi is ideal for the proposed model. The ProSA result obtained a model z-score of −9.05, which is within the range of scores typically found for native proteins of equivalent size in structures obtained via X-ray and NMR (Figure 1C). Additionally, the QMEAN value was 0.34, and the set of Z-values for different parameters, such as C-beta interactions (0.99), interactions between all atoms (0.46), solvation (0.59), and torsion (−0.02), were very close to the value of 0, and this shows the fine quality of the model (Figure 1D). These data confirm the validity of the NTHiENO model as a dimer. 

### 2.2. Molecular Docking of NTHiENO-Collagen

Collagen is composed of three polypeptide chains, called α chains, wound together in a triple helix [16]; type I collagen is typically composed of two α1 chains and one α2 chain [31], while type III collagen is a homotrimer containing three α1 chains [21]. In the PDB database, the three α chains of both collagens are identified as A, B, and C chains. Nevertheless, in this study, to differentiate the A and B chains of NTHiENO, as well as between them, we decided to rename the D chain (α1), E chain (α1′), and F chain (α2) for Cln I (type I collagen) and D chain (α1), E chain (α1′), and F chain (α1″) for Cln III (type III collagen). 

HDOCK was used to search and analyze the possible binding poses between NTHiENO and collagen in 3D space. For NTHiENO-Cln I, the first 10 best coupling results were considered, and it was observed that the binding poses between both proteins were very similar to each other; that is, the residues present in the region of the interface in most cases were maintained in both, for NTHiENO and Cln I; in some predictions, the interface region of the A chain residues participated mainly, and in other predictions, the same residues were identified but of the B chain of the NTHiENO homodimer; however, the contact zone remained in the same region for both proteins in at least 50% of the analyzed models. The best model was selected based on the best docking energy score, which was −249.7. In the case of binding poses between the NTHiENO-Cln III complex, the first 10 best coupling results were also considered. The best docking energy score was −213.22, so this model was selected. 

**Figure 2:**
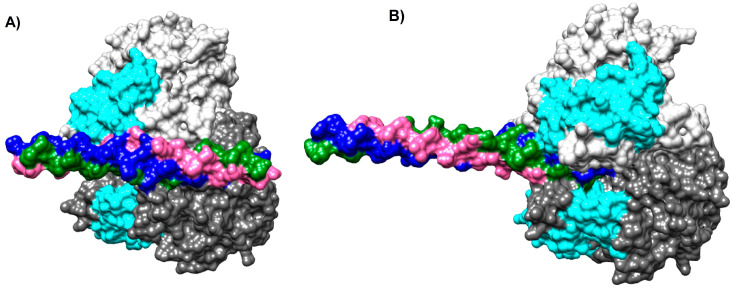
Models of NTHiENO-Collagen complex interaction using HDOCK. Binding proteins are shown as surface representations. (**A**) NTHiENO-Cln I, homo dimer of NTHiENO, is shown in dark grey (A chain), and light grey (B chain); the region of putative binding to collagen for both chains is shown in Cyan, and the ClnI triple helix (7CWK) is shown in pink (α1 chain), blue (α1′ chain), and green (α2 chain). (**B**) NTHiENO-Cln III binding proteins are shown as surface representations. Homo dimer of NTHiENO is in the same order as “A”, and the ClnIII triple helix (6A0A) is shown in pink (α1 chain), blue (α1′ chain), and green (α1″ chain).

These models were visualized on Chimera; in both models, the proteins exhibited surface complementarity, and the putative collagen-binding motif of NTHiENO is present in the interface area (Figure 2A,B). The interface area in Å2 was more with Cln I than Cln III; the results are shown in Table 1.

Moreover, the results obtained via Prodigy showed a binding affinity (kcal/mol) of −10.0 and −5.2, and a Kd (M) at 37.0 °C, of 8.7 × 10^−8^ and 2.3 × 10^−4^, for Cln I and Cln III, respectively. 

### 2.3. Interacting Residues between NTHiENO-Cln I

**Figure 3:**
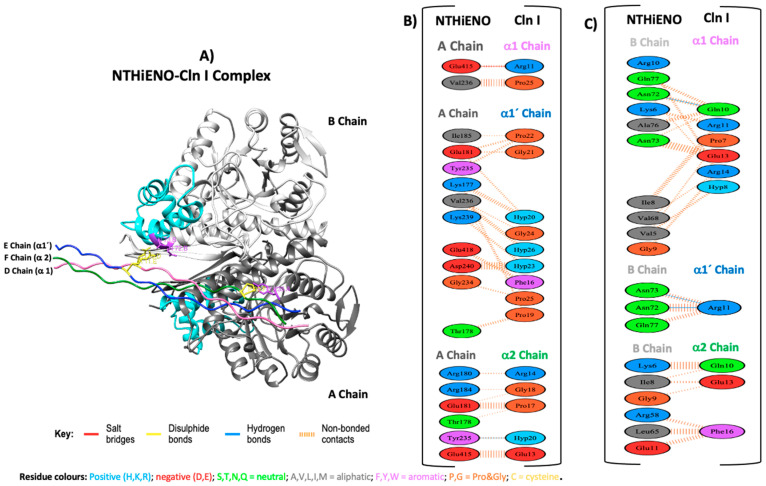
Model of NTHiENO-Cln I complex interaction. (**A**) NTHiENO is shown in dark grey (A chain) and light grey (B chain), while the cyan colour represents the putative collagen-binding region (72–139). The Cln I triple helix (7CWK) is shown in pink (D chain), blue (E chain), and green (F chain), equivalent to α1, α1′ and α2 chains, respectively. The residues involved in the formation of hydrogen bonds are represented as sticks in purple (enolase) and yellow (Cln I). (**B**) PDBsum’s interaction plot for NTHiENO (A chain)-Cln I (α1, α1′, and α2 chains) complex. (**C**) PDBsum’s interaction plot for NTHiENO (B chain)-Cln I (α1, α1′, and α2 chains) complex. The residues are shown in different colours based on their properties, and the coloured lines joining bind these residues represent the type of interaction between them. For non-bonded contacts, the width of the striped line is proportional to the number of atomic contacts. The detail for each type is shown in the figure.

To evaluate the type of interactions and the main residues involved in the interaction from NTHiENO to Cln I, the coupling model was analyzed using the PDBsum platform. The results show that NTHiENO and Cln I maintain conformational stability generated by hydrogen bridges, salt bridges, and other intermolecular forces exerted by multiple residues of both proteins. In the analyses, three important residues in the homodimer of NTHiENO [Tyr235 (A chain), Asn72 y Asn73 (B chain)] and three residues of Cln I [Gln10 (α1 chain), Arg11 (α1′chain) and Hyp20 (α2 chain)] were identified as essential for interaction through the formation of four hydrogen bonds, these residues are shown in the 3D structure of the coupling NTHiENO-Cln I, where it is observed that the residues Asn72 and Asn73 of the B chain belong to the putative region of collagen binding (Figure 3A). In addition, apart from the four hydrogen bonds, the presence of one salt bridge and 194 non-bonded contacts or weak bonds stabilize the interaction between both proteins (Figure 3B,C). The summary of the data is shown in Table 1.

### 2.4. Interacting Residues between NTHiENO-Cln III

**Figure 4:**
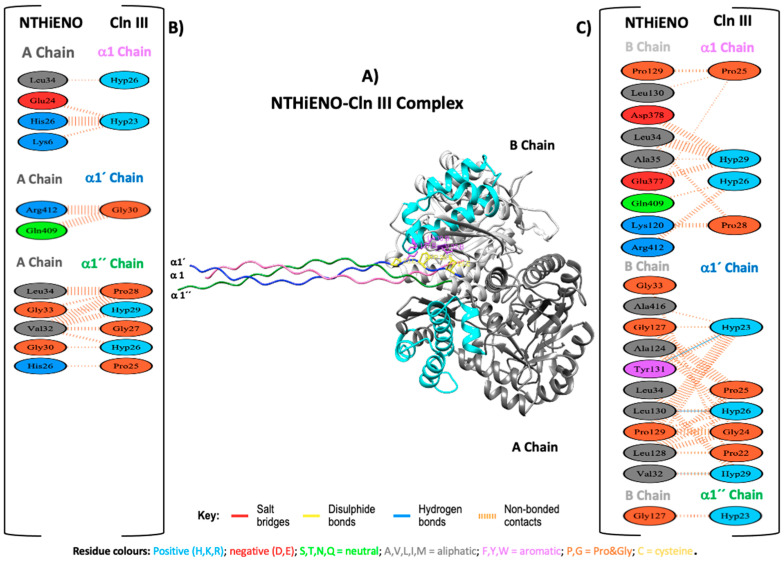
Model of NTHiENO–Cln III complex interaction. (**A**) NTHiENO is shown in dark grey (A chain) and light grey (B chain), while the cyan colour represents the putative collagen-binding region (72–139), and the Cln III triple helix (6A0A) is shown in pink (D chain), blue (E chain), and green (F chain), equivalent to α1, α1′ and α1″ chains, respectively. The residues involved in the formation of hydrogen bonds are represented as sticks in purple (enolase) and yellow (Cln III). (**B**) To the left, PDBsum’s interaction plot for NTHiENO (A chain)-Cln III (α1, α1′ and α1″ chains) complex. (**C**) To the right, PDBsum’s interaction plot for NTHiENO (B chain)-Cln III (α1, α1′ and α1″ chains) complex. The residues are shown in different colours based on their properties, and the coloured lines joining bind these residues represent the type of interaction between them. For non-bonded contacts, the width of the striped line is proportional to the number of atomic contacts. The detail for each type is shown in the figure.

For the interaction of NTHiENO and Cln III, the analysis shows that the binding of both proteins is mediated by three hydrogen bonds and 186 non-bonded contacts or weak bonds (Figure 4B,C); the summary results are shown in Table 2. In this analysis, three important residues of NTHiENO were also identified for the formation of three hydrogen bonds corresponding to Leu130, Tyr131, and Val32 of the B chain and three residues of Cln III corresponding to Hyp23, Hyp26, and Hyp29 of α1′ chain. These residues are localized in the 3D structure of the coupling NTHiENO-Cln III, where it can be observed that the residues Leu130 and Tyr131 of the B chain belong to the putative region of collagen binding (Figure 4A).

### 2.5. Experimental Detection of rNTHiENO-Cln Interaction

Once we identified the type of interactions and the possible NTHiENO residues that could participate in binding to type I and type III human collagen, the next objective was to demonstrate whether such interactions could be observed in vitro assays. To do these assaysm we use purified type I collagen from mouse tail and type III collagen from bovine kidney.

**Figure 5:**
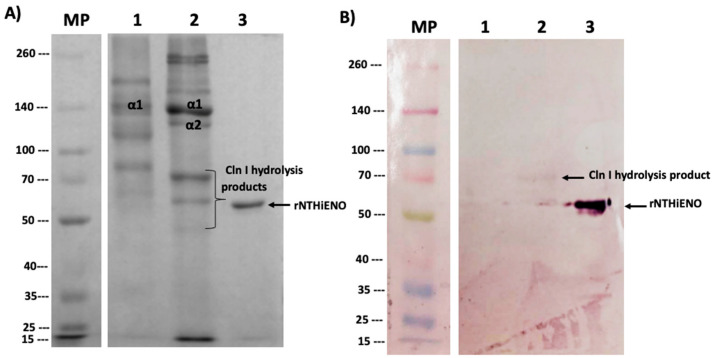
Identification of the binding of rNTHiENO to collagen. (**A**) Protein migration in SDS-PAGE 7.5%. M: weight marker; Lane 1: Cln III; Lane 2: Cln I; Lane 3: rNTHiENO. In the image, the bands corresponding to the α chains are identified as Cln III and Cln I; the not labeled bands represent oligomers of the single α chains. Hydrolyzed peptides of lower molecular weight derived from α chains are also observed. (**B**) Immunodetection of rNTHiENO–Collagen interaction using polyclonal anti-rNTHiENO antibodies as the first antibody, IgG anti-mouse accoupled to alkaline phosphatase was used as the second antibody. After the migrated proteins were transferred to the nitrocellulose membrane and before the use of the antibodies, the blot was incubated with rNTHiENO. M: prestained protein marker; Lanes 1: Cln III; Lane 2: rNTHiENO–Cln I interaction; Lane 3: rNTHiENO.

Far-Western blot analysis was used to study the protein–protein interaction; Cln I (mouse tails), Cln III (bovine kidney), and rNTHiENO were migrated in 7.5% SDS-PAGE. It showed the expected pattern of each of the single-collagen polypeptide chains and a single band for purified rNTHiENO, with an approximate weight of 52 kDa (Figure 5A). The proteins were electrotransferred to a nitrocellulose membrane; it was blocked and later incubated with rNTHiENO. rNTHiENO–Cln I and Cln III interaction was identified using mouse polyclonal antibodies anti-rNTHiENO. The results showed two signals, one corresponding to a fragment of Cln I binding to enolase and another band with lower molecular weight corresponding to rNTHiENO, which was used as a positive control; for Cln III, no binding signal was visualized (Figure 5B); this indicates that rNTHiENO is capable of binding to small fragments of type I collagen α chains; this assay was replicated in independent experiments, and the interaction signal remains constant with Cln I (Supplementary Figure S1). No binding signal was detected to Cln I when the membrane was incubated with BSA as a negative control (Supplementary Figure S2).

**Figure 6:**
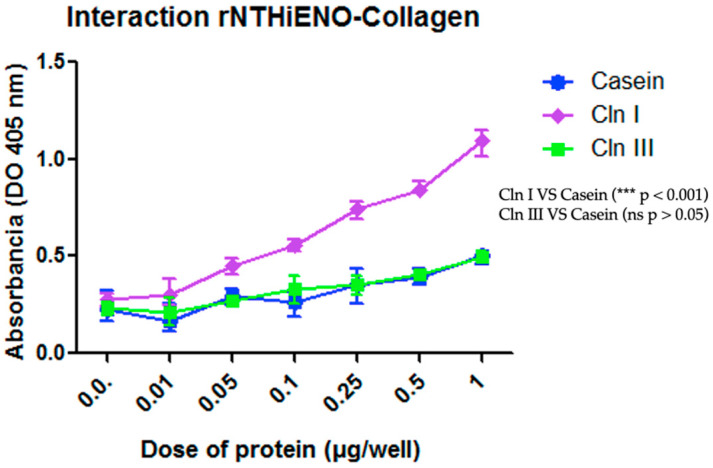
Identification of the binding of rNTHiENO to collagen using ELISA. ELISA plates were coated with 1.0 μg/well of Cln I, Cln III, or casein (negative control) diluted in 100 μL of PBS. rNTHiENO was used with several increased concentrations (0.01–1.0 µg/well), as is shown in the plot. The interaction was quantified by using polyclonal anti-rNTHiENO antibodies as the first antibody and goat anti-mouse IgG-HRP-conjugated as the secondary antibody. The results show that the interaction between rNTHiENO and Cln I is concentration-dependent, while the interaction with Cln III had no significant (ns) difference with respect to the negative control. Data represent the mean ± SD standard deviation of three independent experiments. Statistical significance was performed with the last dose of protein (1 µg) in the experiment (*** *p* < 0.001): Cln I VS Casein and Cln III; (ns): Cln III VS Casein.

In other interaction assays, the results obtained using the ELISA assays showed that rNTHiENO can bound to Cln I in a dose-dependent manner and with statistical significance compared to the negative control (casein), while the interaction with Cln III was not observed and therefore had no significant difference with respect to the negative control (Figure 6).

## 3. Discussion

The extracellular matrix (ECM) makes up a protein complex whose composition and structural organization influence numerous biological processes such as adhesion, migration, proliferation, or differentiation of cells [17,32]. Microorganisms have developed different mechanisms to successfully colonize human tissues. The adhesion to host tissues represents a crucial early step in the colonization process [14]. In the case of *H. influenzae*, some persistence mechanisms include the expression of multiple adhesins that mediate binding to the host´s cells and extracellular matrix components [3]; therefore, the relationship between adhesins and the ECM plays an important role in the success of NTHi colonization and virulence [4].

Enolase is active as a moonlighting protein; the most common moonlighting function of enolases appears to be binding to plasminogen (Plg); several pathogens use the human Plasminogen/Plasmin system to favor their migration across host tissue barriers [9]. In addition, it has also been found to be a cell surface adhesin in a variety of microorganisms interacting with ECM proteins [10,13,14].

In previous works, the presence of enolase on the cell surface of typeable and non-typeable strains of *H. influenzae* was identified [33]; moreover, this protein was identified as a human plasminogen-binding protein [34]. On the other hand, in multiple studies, it has been reported that among the components of the ECM, collagen is one of the targets of interaction with the enolase of many pathogens and some prebiotic bacteria [9,25,26,27]. However, to date, in the case of *H. influenzae* enolase, there are no reported data. Therefore, in this study, we have characterized the ability of rNTHiENO as a type I and III collagen-binding protein with both in silico and experimental tests.

Until now, the crystallized structure of *H. influenzae* enolase is not available in the databases; however, crystallized structures of enolase from other organisms are available. Therefore, we proceeded to obtain the structure of NTHiENO through homology modeling. *E. coli* enolase homo dimer was used as a template with a resolution value of 1.81 Å; both proteins presented a high percentage of identity (85.61%), thereby supplying a suitable template for the modeling of NTHiENO. The stereochemical quality of the NTHiENO structure was verified via the PROCHECK Ramachandran plot, showing that the position of the amino acid residues is within the most favored or allowed regions with a value of 99.5%; the Z-score calculated (−9.05) using the ProSA server is in the range of native protein conformation scores. The normalized QMEAN value was 0.34, suggesting that the model obtained does not differ significantly from the experimental structures; the quality estimate ranges are between 0 and 1 [35]; therefore, our model was within the typical standard deviation value. These data indicate that the modeling is reliable.

Type I collagen is found in all extracellular matrices, including bone, skin, and tendons, while Type III collagen is also an important component of blood vessels and hollow organs [36]; both are the major constituents of the ECM [37], belong to large fibrillar collagens, and can frequently be found together [36]. 

A study conducted by Antikainen et al. in 2007 shows that the enolase of *Staphylococcus aureus* and *Lactobacillus crispatus* present a strong binding to type I collagen [12]; this was also observed in *Lactobacillus casei* BL23 [38], *Paracoccidioides brasiliensis* [26] and *Lactobacillus plantarum*; interestingly, in this last study, the authors reported that, by using truncated recombinant enolase proteins, they showed that the region spanning from 73 to the 140 amino acid residues is involved in type I collagen binding, additionally, they observed that the mutant strain LM3-CC1, carrying a null mutation in the enoA1 gene, binds to immobilized collagen less efficiently than the wild-type strain and that purified EnoA1 can bind to collagen under both denaturing and native conditions [39]. Comparable results in terms of adhesion were seen with the recombinant enolase of *Staphylococcus lugdunensis* (rSIEno) binding to type IV collagen [9]; these results are comparable to those obtained in this work, where the interaction between type I collagen and NTHiENO is determined, demonstrating that it is dose-dependent and with statistical significance when compared with the casein control.

In the results obtained using HDOCK, we observed that NTHiENO presents surface complementarity with both Cln I and Cln III; for both cases, multiple models were analyzed, and approximately 50% of the predictions of coupling poses for each collagen occur in the same region. Nevertheless, the main residues belonging to chain A or chain B of NTHiENO are present in the interface region, indicating that the interaction may be mediated by both chains (A and B), but the NTHiENO–Collagen complex can be carried out mainly with the A chain or with the B chain of *H. influenzae* enolase; these results are not surprising, since as it is a homodimer and both chains have the same sequence. The best model was selected based on the best docking energy score, which was −249.7 and −213.22 for Cln I and Cln III, respectively. In both cases, the putative collagen-binding motif of NTHiENO (72–139) is equivalent to the region identified as involved in type I collagen binding (73–140) of *L. plantarum* enolase, and this region is present in the interface area; however, for the case of NTHiENO–Cln I interaction, interphase Area (Å2) was greater concerning NTHiENO-Cln III (see Table 1 and Table 2).

On the other hand, the analysis performed with PDBsum of complex NTHiENO-Cln I identified three important residues in the homodimer of NTHiENO [Tyr235 (A chain), Asn72 y Asn73 (B chain)], involved in the formation of four hydrogen bonds with Cln I; two of them Asn72 y Asn73 (B chain) interact with Gln10 and Arg 11 of α1 chains of Cln I by forming three hydrogen bonds, and the third Tyr235 (A chain) interacts with the Hyp20 of α2 chain of Cln I. In addition, a salt bridge between Glu415 (Chain A) and Arg 11 α1 chain was also identified (Figure 3B,C). These results could indicate that NTHiENO presents better affinity with the α1 chains with respect to the chain α2 of Cln I due to the formation of hydrogen bonds in addition to the multiple weak bonds. Interestingly, these results were visualized in the far-Western blot assays, where it was observed that rNTHiENO interacts with a hydrolyzed peptide (small fragment) of Cln I α chains (Figure 5B). The ability of rNTHiENO to bind Cln I was also examined using ELISA assays, and the results show that rNTHiENO binds to Cln I in a concentration-dependent manner (Figure 6). These residues could be very important in the interaction with NTHiENO, given that, in multiple studies, the role of Hyp is highlighted by participating in specific interactions with other biomolecules [23,40].

In the case of the analysis of interactions of the NTHiENO-Cln III complex, it was observed that the interaction could be mediated mainly by three hydrogen bonds corresponding to Leu130, Tyr131, and Val32 of B chain of NTHiENO and three residues of Cln III corresponding to Hyp23, Hyp26, Hyp29 of α1’ chain, in addition to multiple weak interactions. However, in the far-Western blot tests, no signal was observed, as well as in the Elisa test, the ability of rNTHiENO to bind to Cln III observed in the interaction kinetics did not have a significant difference concerning the casein protein that was used as a negative control (Figure 6). It should be remembered that molecular docking was conducted with the crystallized structure of human type III collagen, and in the in vitro tests, type III bovine collagen was used. We suppose that this lack of interaction observed in vitro assays could be due to the tissue tropism that presents *H. influenzae* to its host since it exclusively colonizes the human nasopharynx [41].

Type I collagen was purified from the mouse tail, and with this collagen, in vitro interaction with NTHiENO was observed. To confirm this result, alignments with sequences for type I human collagen and mouse type I collagen were performed, and the results showed a high percentage of identity: 92.36%thus, this high degree of similarity allows us to extrapolate the results what would be happening naturally with human type I collagen, wherein rNTHiENO could interact with human type I collagen, and this interaction probably would be mainly given with the α1 chains, as demonstrated through the in silico approach using molecular docking.

However, based on these results, we can hypothesize that there is a high probability of the interaction of NTHiENO with human collagen type III. Although the affinity would be preferentially towards type I collagen, as observed in the results obtained using Prodigy, where the values of the Gibbs free energy ΔG: −10.0, as well as the dissociation constant Kd: 8.7 × 10^−8^ observed, were better with respect to values obtained for type III collagen ΔG: −5.2 and Kd: 2.3 × 10^−4^; moreover, Cln I also presented the highest formation of hydrogen bonds, salt bridges and weak bonds, and these results were consistent with the in vitro assays; therefore, we conclude that NTHiENO could present a better ability to bind to Cln I than to Cln III human. This has been reported in studies with enolases of *S. aureus* and *L. crispatus* [12], *L. casei* BL23 [38], *P. brasiliensis* [26] and *L. plantarum* [39], which has a strong binding to type I collagen.

The better affinity that exists towards collagen type I concerning collagen type III may probably be due to the differences in the structure that they present, taking into account that the triple helix of type-I collagen is composed of two α1(I) chains and one α2(I) chain [19], while type III collagen is a homotrimer of three identical polypeptide chains [20] supercoiled around each other in a right-handed triple helix [21], we assume that exposure of certain residues favors better interaction with Cln I. Interestingly, some of the amino acids of Cln I involved in the interaction with NTHiENO are found within the GQRGER motif, which has been reported as a functional interaction motif with other proteins [23,42], so the best affinity of NTHiENO towards Cln I also could be due to this motif

As already mentioned above, *L. plantarum* enolase showed that the region spanning from 73–140 amino acid residues is involved in type I collagen-binding [39]. In our results, the residues Asn72 Asn73 (B chain) and Tyr235 (A chain), and Leu130, Tyr131(B chain) of homodimer of NTHiENO, essential for the formation of hydrogen bonds with collagen type I and collagen type III, respectively, and moreover, these amino acids are present in the putative collagen-binding motif of NTHiENO (72–139) (Figure 3A and Figure 4A). Therefore, with this antecedent and the results obtained in this work, we propose (Asn72 y Asn73) and (Leu130 and Tyr131) residues of NTHiENO as the main binding sites to Cln I and ClnIII, respectively; however, other residues could be been the vital important maintaining a proper folding of NTHiENO that is favorable for their interaction with both Cln I or Cln III, but this hypothesis will have to be confirmed in further studies.

Thereby, human collagen could be an important target for enolase of both typeable and non-typeable *H. influenzae* strains (Due to the high percentage of identity that they present between them 99.54%) [33]; this interaction could enhance bacterial adhesion to one of the main components of the ECM, and therefore promote the invasion of host cells, which could be favored by its interaction whit human plasminogen (Plg) and its subsequent conversion into plasmin (Plm) [43], a serine protease that promotes the degradation of the extracellular matrix; this mechanism is used by some pathogens to infect and invade host cells [44].

## 4. Materials and Methods

### 4.1. Homology Modeling of NTHiENO Dimer

Modeling via the homology of non-typeable *H. influenzae* dimeric enolase (NTHiENO) (GenBank: MF405339) was performed with the SWISS-MODEL platform (Swiss Institute of Bioinformatics, Biozentrum, University of Basel Spitalstrasse, 41CH-4056 Basel/Switzerland) [28], using as a template the crystallized structure of *E. coli* enolase (PDB ID 6BFY) [30]. Subsequently, the quality and stereochemistry were evaluated using the PROCHECK program (Genome Campus, Hinxton, Cambridgeshire, CB10 1SD, UK [45], the ProSA server (Protein Structure Assessment) [46], and the QMEAN (Qualitative Model Energy Analysis) [35].

### 4.2. Analyses of Protein–Protein Docking

The crystallized structures of human type I collagen (Cln I) PDB ID (7CWK) (RCSB PDB—7CWK: Structure of a triple-helix region of human collagen type I, n.d.), and human type III collagen (Cln III) PDB ID (6A0A) [47] were obtained from the Protein Data Bank. The structures of NTHiENO, Cln I and Cln III were first prepared with the Chimera program [29], and later the protein–protein docking was elaborated using an online version of the HDOCK server [48]. The first 10 best coupling results were analyzed, and the one with the best docking energy score was selected for both cases. The binding mode was visualized by Chimera [29], and the analysis and visualization of the interaction protein–protein were performed using PDBsum (Genome Campus, Hinxton, Cambridgeshire, CB10 1SD, UK) [49]. Finally, the select models were submitted for analysis to evaluate the binding affinity (∆G) and dissociation constant (Kd) using the PRODIGY platform [50].

### 4.3. Far-Western Blot Assay

To detect the binding between rNTHiENO-Cln I and rNTHiENO-Cln III, far-Western Blot assays were performed as described by Hall [51]. Briefly, 3 μg of the Cln I (mouse tail) or 3 μg of the Cln III (bovine kidney), purified as described previously [52], and 3 μg rNTHiENO purified as described [33] were migrated in SDS-PAGE (7.5%), and electrotransferred onto a nitrocellulose membrane (Bio-Rad, Inc., Hercules, CA, USA). The membranes were incubated for 1 h/RT using blocking buffer (2% non-fat powdered milk, 0.1% Tween-20 in PBS) and were incubated overnight at 4 °C with 10 μg/mL of rNTHiENO or 10 μg/mL of BSA (negative control) in blocking buffer. After three times of washing with blocking buffer, the membranes were incubated with polyclonal anti-rNTHiENO antibodies as the first antibody and with goat anti-mouse IgG alkaline phosphatase-conjugated as the secondary antibody (Novex^®^ by Life Technologies, Van Allen Way, Carlsbad, CA, USA). The signals were visualized using NBT (nitro blue tetrazolium) and BCIP (5-Bromo-4-chloro-3-indolyl-phosphate) (Thermo Fisher Scientific, Waltham, MA, USA).

### 4.4. ELISA Assays

Detection of Interactions of rNTHiENO with collagen was assayed using enzyme-linked immunosorbent assay (ELISA). Briefly, individual wells of micro-ELISA plates were coated with 1.0 μg/well of Cln I or Cln III diluted in 100 μL of PBS (phosphate-buffered saline) or with casein (as a negative control) at the same concentration and were incubated at 4 °C overnight. The plates were washed three times with PBS-Tween 0.5% (PBST). After that, the wells were blocked with 3% casein for 2 h at 37 °C, followed by washing three times again and incubated with 0.01–1 μg/well of rNTHiENOdiluted in 100 μL of PBS, and were incubated at 4 °C overnight. The binding protein was detected with polyclonal anti-rNTHiENO antibodies as the first antibody and goat anti-mouse IgG-HRP-conjugated as the secondary antibody. The interaction was visualized using 3,3′,5,5′-Tetramethylbenzidine (TMB; Sigma-Aldrich, Gewerbegebiet Süd, Kappelweg 1, 91625 Schnelldorf, Germany), and the reaction was stopped with the addition of 50 μL of 0.5 M sulfuric acid, and, finally, the OD was read at 450 nm. All experiments were repeated three times independently.

## 5. Conclusions

The data presented in this work indicate that recombinant enolase of non-typeable *H. influenzae* is capable of binding type I collagen in a concentration-dependent manner, and this interaction could be associated principally with α1 chains of Cln I. Moreover, we identified amino acids with a high probability of being involved in the interaction of NTHiENO-Cln I and NTHiENO-Cln III. Finally, we observed that NTHiENO showed a better affinity to interact with the type I collagen with respect to type III. This interaction could have a relationship with the pathophysiology of *H. influenzae*, since by interacting with human collagen, NTHiENO could improve the adhesion and invasiveness capacity of *H. influenzae* and determine the course of infections associated with this bacterium. Nevertheless, it is necessary to carry out more studies to corroborate our hypotheses. However, the results obtained in this work provide an important guideline to continue investigating the role of NTHiENO as a determinant virulence factor.

## Figures and Tables

**Table 1:** Summary results of NTHiENO- Cln I interactions.

Chains NTHiENO-Cln I	Interface Residues	Interface Area (Å2)	Salt Bridges	Disulphide Bonds	Hydrogen Bonds	Non-Bonded Contacts
A–D (α1)	2:2	142:164	1	-	-	5
A–E (α1′)	10:9	423:476	-	-	-	73
A–F (α2)	6:5	364:394	-	-	1	36
B–D (α1)	10:6	408:511	-	-	1	37
B–E (α1′)	3:1	105:146	-	-	2	18
B–F (α2)	6:3	212:294	-	-	-	25

**Table 2:** Summary results of NTHiENO–Cln III interactions.

Chains NTHiENO-Cln III	Interface Residues	Interface Area (Å2)	Salt Bridges	Disulphide Bonds	Hydrogen Bonds	Non-Bonded Contacts
A–D (α1)	4:2	145:190	-	-	-	17
A–E (α1′)	2:1	67:91	-	-	-	9
A–F (α1″)	5:5	228:285	-	-	-	30
B–D (α1)	9:4	292:430	-	-	-	61
B–E (α1′)	10:6	325:412	-	-	3	68
B–F (α1″)	1:1	38:37	-	-	-	1

## Data Availability

The data presented in this study are available upon request from the corresponding author.

## Supplementary Materials

The following supporting information can be downloaded at: https://www.mdpi.com/article/10.3390/ijms242115499/s1.
