# Supplementary figures and images for: The Enolase of the Haemophilus influenzae Mediates Binding to Collagens: An Extracellular Matrix Component
Source: Int J Mol Sci. 2023 Oct 24;24(21):15499. doi: 10.3390/ijms242115499 (PMC10650631; doi:10.3390/ijms242115499)

## Slide 1
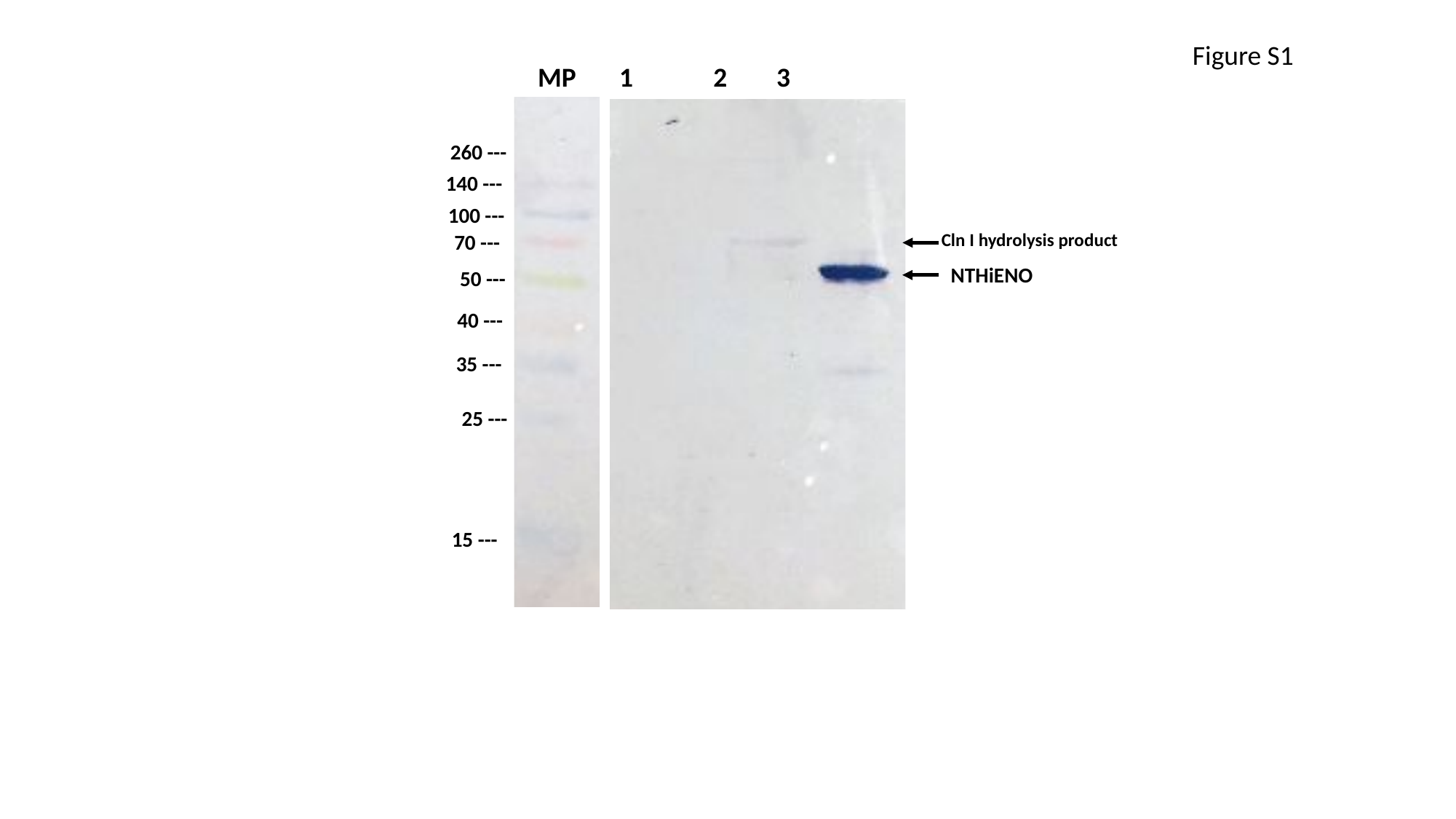

Figure S1
 MP 1 2 3
260 ---
140 ---
70 ---
50 ---
40 ---
35 ---
25 ---
15 ---
100 ---
Cln I hydrolysis product
NTHiENO

## Slide 2
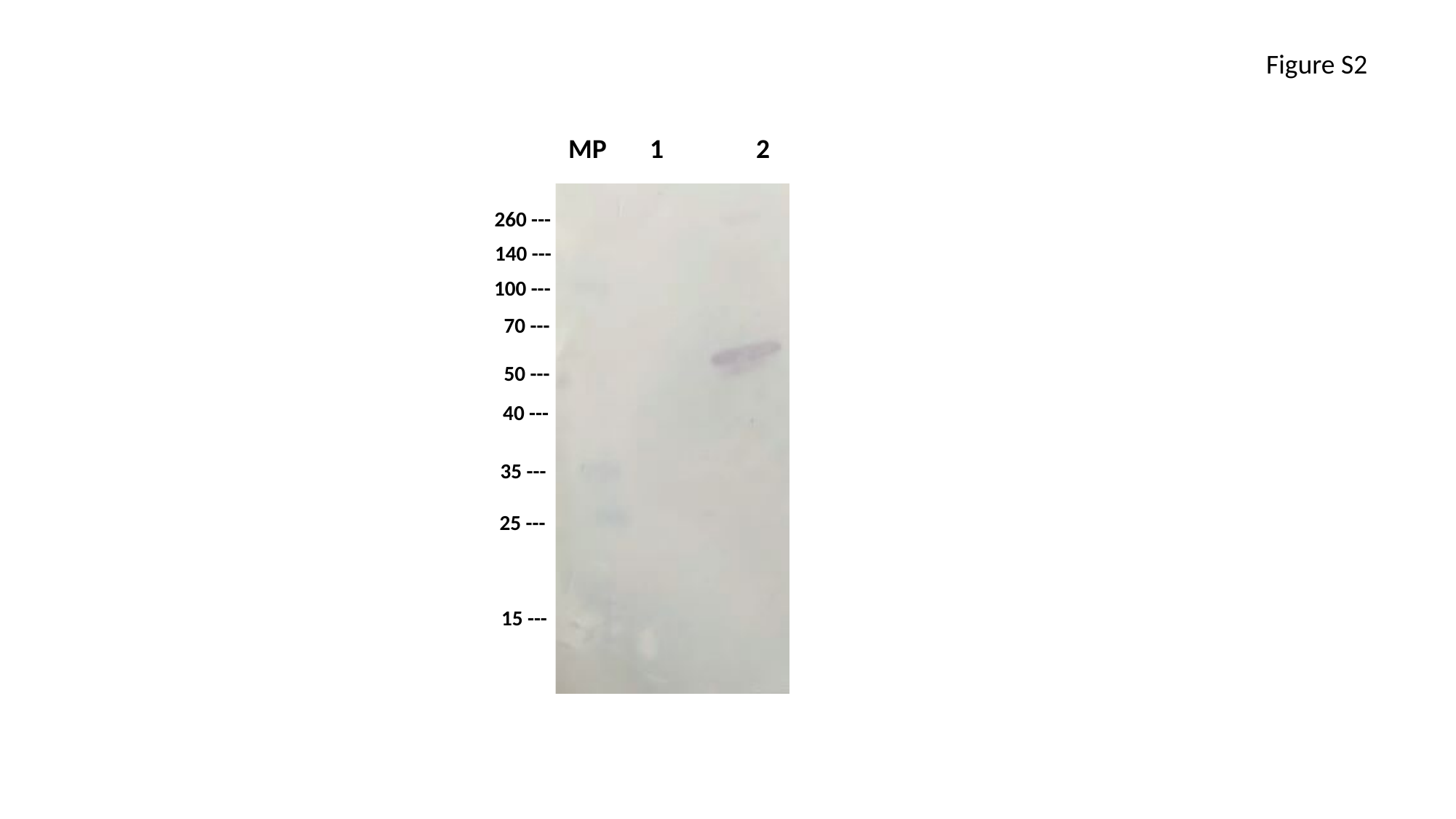

Figure S2
MP 1 2
25 ---
15 ---
35 ---
50 ---
140 ---
100 ---
70 ---
40 ---
260 ---

Supplement: Supplementary file 1 [file ijms-24-15499-s001.zip › ijms-2575747-supplementary.pptx]
